# Comparison of Serum Vitamin B12 Levels Among Drug-Naïve and Metformin-Treated Patients With Polycystic Ovary Syndrome

**DOI:** 10.7759/cureus.30447

**Published:** 2022-10-18

**Authors:** ABM Kamrul-Hasan, Fatema Tuz Zahura Aalpona

**Affiliations:** 1 Endocrinology, Mymensingh Medical College, Mymensingh, BGD; 2 Obstetrics and Gynecology, Mymensingh Medical College, Mymensingh, BGD

**Keywords:** cobalamin deficiency, anemia, vitamin b12, metformin, pcos

## Abstract

Background

Metformin is frequently prescribed for polycystic ovary syndrome (PCOS). There is a well-established causal relationship between metformin use in type 2 diabetes and vitamin B12 deficiency; such a relationship is seldom studied in PCOS. We conducted this study to compare vitamin B12 levels among newly diagnosed and metformin-treated patients with PCOS.

Methodology

This cross‑sectional comparative study was conducted from July 2021 to June 2022, among patients with PCOS attending a specialized endocrinology clinic of a tertiary hospital in Mymensingh, Bangladesh. A total of 50 patients newly diagnosed with PCOS and 52 with PCOS who had received metformin for at least six months were evaluated. The serum vitamin B12 level was measured by utilizing the chemiluminescent microparticle immunoassay principle.

Results

The new drug-naïve and metformin-treated subjects with PCOS had similar clinical and laboratory parameters except for the metformin group’s lower hemoglobin levels and higher plateletcrit. Metformin receivers had lower serum vitamin B12 levels than the drug-naïve subjects (385.5 pg/mL [interquartile range, or IQR, 298.7-535.2] vs. 272.0 pg/mL [IQR 217.0-395.7]; *P *< 0.001). The metformin group had higher frequencies of B12 deficiency and borderline deficiency (15.4% vs. 6% and 42.3% vs. 18%, respectively; *P *= 0.003).

Conclusions

This study observed lower serum B12 levels in PCOS patients using metformin than in the newly diagnosed ones. Large-scale data are needed to recommend routine periodic screening for B12 levels in metformin-treated PCOS.

## Introduction

Polycystic ovary syndrome (PCOS) is a common endocrine disorder affecting 6% to 12% of women of reproductive age worldwide [1]. PCOS is a complex and heterogeneous disorder with diverse clinical manifestations, including menstrual irregularities, hyperandrogenism, and polycystic ovarian morphology in imaging studies. Although its exact etiology remains elusive, PCOS features several hormonal disturbances, including hyperandrogenemia, insulin resistance, and hyperinsulinemia [2]. Insulin resistance and hyperinsulinemia play critical roles in the molecular mechanisms implicated in the androgenic hypersecretion typical of PCOS pathology [3,4]. PCOS increases the risk of diabetes and glucose intolerance independent of obesity [5,6]. Insulin-sensitizing pharmacotherapy, including metformin, concurrently lowers androgenemia and improves ovarian functionalism [4]. Insulin resistance and the high risk of hyperglycemia have underpinned the use of metformin in PCOS. Moreover, metformin is an excellent adjunctive medication for ovulation induction in women with PCOS for high- and low-complexity-assisted reproduction therapies [7]. Although most pharmacological treatments, including metformin, are generally off-label in PCOS and there is variability in recommendations across health professional specialties, metformin is widely prescribed and used by women with PCOS [8,9].

Metformin use appears safe; however, its long-term use could reduce vitamin B12 levels in a dose-dependent manner [10]. Vitamin B12 (cobalamin) is a vital nutrient for health, and its deficiency may have adverse hematological, neuropsychiatric, and cardiovascular consequences [10]. Although data regarding B12 deficiency in metformin-treated patients with type 2 diabetes mellitus (T2DM) are widely available in the literature, similar evidence in PCOS is scarce [10,11]. With this background, we conducted this study to compare vitamin B12 levels among newly diagnosed and metformin-treated patients with PCOS.

## Materials and methods

This cross‑sectional comparative study was conducted from July 2021 to June 2022 among patients with PCOS attending a specialized endocrinology clinic of a tertiary hospital in Mymensingh, Bangladesh. A total of 50 patients newly diagnosed with PCOS and 52 with PCOS who had received metformin for at least six months were evaluated. PCOS was diagnosed using the revised Rotterdam criteria for adults [12]. In adolescent girls, PCOS diagnosis was made based on clinical and/or biochemical hyperandrogenism (after the exclusion of other causes) in the presence of persistent menstrual irregularities [13]. Patients having any comorbid conditions that may interfere with the vitamin B12 level (e.g., pernicious anemia, malabsorption, and gastrointestinal surgery), those with thyroid disorders and advanced hepatic and renal disease, those with chronic (three months or more) use of acid suppressants (i.e., proton pump inhibitors or H2 receptor blockers) and chronic alcohol abuse, and those who are vegetarians, getting supplementation of B12 or any B12 containing multivitamin, and using drugs that affect B12 levels (e.g., corticosteroids, phenytoin, and dihydrofolate reductase inhibitors) were excluded. Informed written consent was taken from the study subjects. The study was conducted according to Good Clinical Practice and the Declaration of Helsinki.

A convenient sampling technique was followed to collect samples. The study subjects were divided into two groups: those with ongoing treatment with metformin for at least six months were included in the metformin group, and those newly diagnosed with PCOS and who never received metformin in their lifetime were included in the non-metformin group. A semistructured questionnaire-based interview was conducted to collect relevant demographic and clinical information. Random venous blood samples were collected, and complete blood count (CBC) and serum vitamin B12 levels were assessed on the same day of blood sample collection. Using the Fluorescence Flow Cytometry method, the CBC was estimated by the hematology autoanalyzer Sysmex XN-2000 (Sysmex, Kobe, Japan). The serum vitamin B12 level was measured by an automated immunoassay analyzer Alinity I System (Abbott Laboratories, Abbott Park, IL, USA) utilizing the chemiluminescent microparticle immunoassay (CMIA) principle. The serum vitamin B12 level ≤200 pg/mL was labeled as a deficiency, >200 to ≤300 pg/mL as a borderline deficiency, and >300 pg/mL was labeled as normal [14].

Data were analyzed using the IBM SPSS Statistics for Macintosh software (Version 28.0; IBM Corp. Released 2021, Armonk, NY, USA). The continuous variables with and without a normal distribution were expressed as mean ± standard deviation and median (interquartile range [IQR]), respectively. The categorical variables were presented as the percentage (number). As applicable, the Student’s t-test, the Chi-square test, and the Mann-Whitney U test were applied to compare the variables between the two groups. A two-sided *P-*value of less than 0.05 indicates statistical significance.

## Results

A total of 50 drug-naïve women newly diagnosed with PCOS and 52 women previously diagnosed with PCOS and on metformin were evaluated; the sample size gave 98.7% power to the study. In the metformin group, the median duration of metformin use was 13.5 (IQR 7.0-23.5) months and the median metformin dose was 1.0 (IQR 0.78-1.0) g.

Women in the two groups had similar age; BMI; systolic and diastolic blood pressure (BP) readings; fasting plasma glucose (FPG); WBC; neutrophil, lymphocyte, monocyte, eosinophil, and red blood cell (RBC) counts; mean corpuscular volume (MCV); mean corpuscular hemoglobin (MCH); MCH concentration (MCHC); red cell distribution width-coefficient of variation (RDW-CV); mean platelet volume (MPV); platelet distribution width (PDW); and erythrocyte sedimentation rate (ESR). The metformin group had lower hemoglobin levels and higher plateletcrit (PCT) than drug-naïve subjects (Table 1).

**Table 1:** Comparison of clinical and laboratory parameters of new drug-naïve and metformin-treated subjects with PCOS. BMI, body mass index; BP, blood pressure; ESR, erythrocyte sedimentation rate; FPG, fasting plasma glucose; MCV, mean corpuscular volume; MCH, mean corpuscular hemoglobin; MCHC, mean corpuscular hemoglobin concentration; MPV, mean platelet volume; PCT, plateletcrit; PCOS, polycystic ovary syndrome; PDW, platelet distribution width; RBC, red blood cell; RDW-CV, red cell distribution width-coefficient of variation; WBC, white blood cell

Variables	Drug-naïve (*n* = 50)	Metformin-treated (*n* = 52)	*P*-value
Age (years)	21.0 (18.7-25.0)	22.5 (19.0-26.0)	0.221
BMI (kg/m^2^)	26.38 (23.41-29.12)	25.13 (22.77-27.26)	0.123
Systolic BP (mmHg)	125 (115-130)	120 (110-130)	0.442
Diastolic BP (mmHg)	80 (75-80)	80 (71-80)	0.910
FPG (mmol/L)	4.9 (4.6-5.4)	4.8 (4.5-5.2)	0.542
WBC (K/mL)	9.24 ± 2.38	9.61 ± 2.07	0.411
Neutrophil (K/mL)	5.46 ± 1.77	5.48 ± 1.63	0.944
Lymphocyte (K/mL)	3.12 ± 1.02	3.40 ± 0.86	0.132
Monocyte (K/mL)	0.36 (0.29-0.47)	0.35 (0.30-0.44)	0.920
Eosinophil (K/mL)	0.23 (0.13-0.36)	0.28 (0.17-0.42)	0.149
RBC (K/mL)	4.84 (4.36-5.11)	4.62 (4.30-4.84)	0.079
Hemoglobin (g/dL)	12.85 (12.0-13.52)	12.45 (11.45-13.18)	0.048
Hematocrit (%)	38.95 (36.07-41.42)	38.3 (35.5-40.3)	0.158
MCV (fL)	82.2 (77.3-87.8)	83.1 (77.9-86.7)	0.883
MCH (pg)	27.4 (25.9-29.1)	27.6 (25.3-28.9)	0.673
MCHC (g/dL)	32.9 (32.1-34.2)	32.5 (32.0-33.3)	0.115
RDW-CV (%)	13.1 (12.6-13.9)	13.2 (12.5-14.3)	0.653
Platelet (K/mL)	306.5 (262.7-354.7)	325.5 (291.0-381.5)	0.074
MPV (fL)	10.3 (9.8-11.0)	10.3 (9.8-11.3)	0.889
PCT (%)	0.33 ± 0.06	0.345 ± 0.06	0.031
PDW (fL)	12.8 (11.1-14.8)	12.0 (10.5-14.2)	0.236
ESR (mm in the first hour)	25.0 (13.2-33.2)	25.0 (17.0-35.7)	0.357

**Figure 1:**
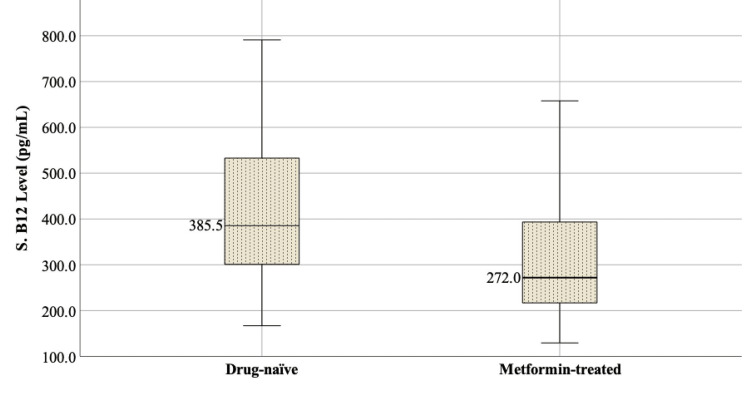
Comparison of vitamin B12 levels among drug-naïve and metformin-treated subjects with PCOS. PCOS, polycystic ovary syndrome

Metformin receivers had lower serum vitamin B12 levels than the drug-naïve group [385.5 (IQR 298.7-535.2) pg/mL vs. 272.0 pg/mL (IQR 217.0-395.7), *P *< 0.001] (Figure 1).

**Figure 2:**
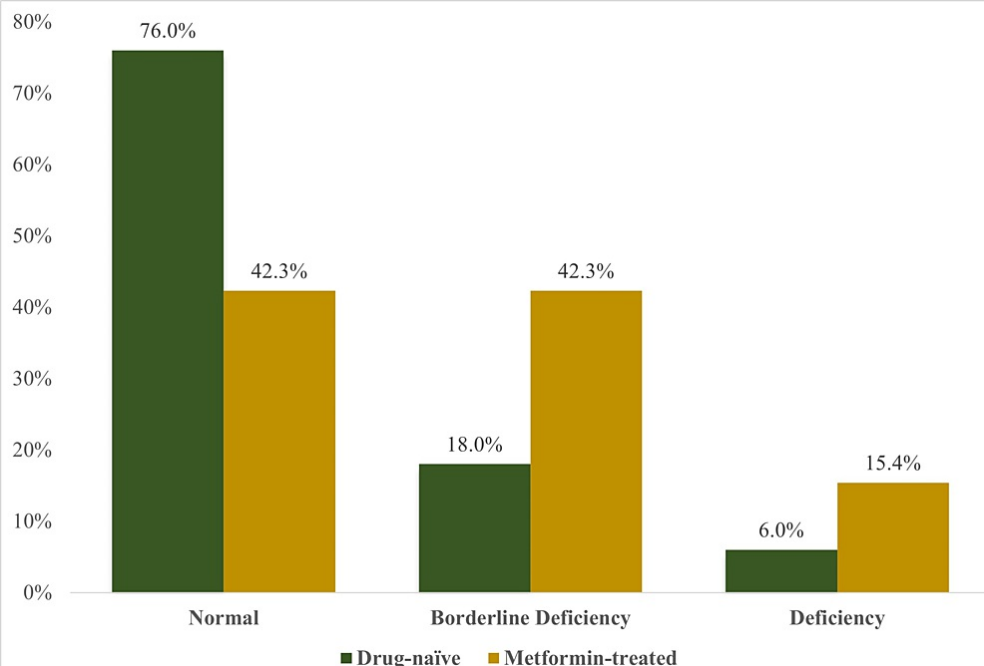
Comparison of vitamin B12 status among drug-naïve and metformin-treated subjects with PCOS. PCOS, polycystic ovary syndrome

The frequency of B12 deficiency and borderline deficiency was higher in the metformin group (*P *= 0.003; Figure 2).

## Discussion

This study investigates the vitamin B12 status in metformin-treated patients with PCOS and compares their B12 levels with those of newly diagnosed, drug-naïve patients with PCOS. We observed that metformin receivers had lower serum vitamin B12 levels than the drug-naïve group and that the frequency of B12 deficiency and borderline deficiency was higher in the metformin group. Metformin receivers also had lower hemoglobin levels than the drug-naïve group.

Vitamin B12, or cobalamin, is a vital nutrient for health that plays an essential role in the functioning of the nervous system and the formation of RBCs. In addition to anemia, B12 deficiency may lead to peripheral neuropathy and dementia [15]. Furthermore, B12 participates in the homocysteine metabolism pathway, and B12 deficiency leads to hyperhomocysteinemia, which is strongly linked to cardiovascular disease [16]. Metformin is a commonly prescribed drug for PCOS. Generally, metformin is well tolerated except for gastrointestinal intolerance in some patients. Metformin use, especially in the long term, is associated with vitamin B12 deficiency [17]. Competitive inhibition or inactivation of B12 absorption; alterations in intrinsic factor levels; gut bacterial flora; gastrointestinal motility, or ileal morphological structure; interaction with the cubulinendocytic receptor; and impairment of calcium-dependent membrane activity in the ileum, including uptake of the B12-intrinsic factor complex, are the postulated mechanisms of metformin-induced B12 deficiency [11]. Moreover, in one study, serum vitamin B12 concentrations were significantly lower in obese PCOS women compared to obese control women (227.8 ± 99.2 vs. 317.6 ± 69.2; *P* < 0.05); such baseline deficit may amplify the risk of B12 depletion in these patients when they are treated with metformin [18].

Metformin-associated B12 deficiency in T2DM was first described in 1969, and since then, many researchers have confirmed the dose-dependent lowering of B12 levels with metformin [10,11,19]. Only a few studies have evaluated the changes in vitamin B12 in metformin-treated patients with PCOS, and there is great heterogenicity among the study results. Kilicdag et al. observed a nonsignificant reduction of vitamin B12 after treatment with 1.7 g daily metformin for three months (281.83 ± 24.51 to 226.08 ± 35.42 pg/mL; *P* = NS) [20]. In another study, Kilicdag et al. explored a nonsignificant increase in B12 (288.68 ± 24.10 to 328.56 ± 136.81 pg/mL; *P* = NS) after 1.7 g daily metformin for three months [21]. In lean patients with PCOS, 1.7 g daily metformin for 12 weeks was associated with a nonsignificant reduction in vitamin B12 levels (311.19 ± 71.74 to 309.23 ± 71.12 pg/mL; *P *= NS) in a study by Yilmaz et al. [22]. On the contrary, Esmaeilzadeh et al. found a significant decrease in the mean B12 level in patients after six months of metformin 1 g/day treatment (456.88 ± 172.32 to 367.69 ± 28.10 pg/mL; *P *= 0.002) [23]. In a meta-analysis, including these four studies, Li et al. showed no significant change in vitamin B12 levels before and after metformin treatment (mean difference 24.70 pg/mL; 95% confidence interval [CI] −22.54 to 71.93; *P *= 0.31; moderate heterogeneity [*I*^2^ = 74%]) [24]. Treatment with metformin was followed by a decline in B12 levels in studies by Greibe et al. (1.5-2.5 g/day for six months) and Khalil et al. (1.5 g/day for 16 weeks) [25,26]. Palmoba et al. observed no significant reduction in B12 levels (379.3 ± 108.1 to 364.7 ± 123.4; *P *= NS) in PCOS patients treated with 1.7 g daily metformin for six months [27]. We found that 24% of the newly diagnosed women with PCOS have subnormal B12 levels, which supports Kaya et al.’s finding of lower B12 levels in PCOS than in healthy controls [18]. Though we did not observe the temporal changes in B12 levels in our study subjects, comparison with a control group with similar characteristics justifies our findings of lower B12 levels in metformin users.

The available evidence on changes in B12 levels with metformin in PCOS is inconclusive. Moreover, despite a reduction in total B12 levels with metformin, a study reported no reduction in the active physiological part of cobalamin bound to transcobalamin (holotranscobalamin) or increase in the metabolic marker of cobalamin status, methylmalonic acid. Instead, the nonfunctional part of circulating cobalamin bound to haptocorrin declined [25]. Due to unavailability, we could not assess holotranscobalamin and methylmalonic acid in our study subjects.

This study has many limitations. It was a single-center study with a small sample size questioning the generalizability of the study result. Due to the cross-sectional design and lack of a healthy control group, the causality assessment for B12 deficiency with metformin was beyond our scope. Measurement of holotranscobalamin and methylmalonic acid would strengthen our findings. As we did not assess folic acid and homocysteine levels, the effect of B12 reductions could not be evaluated. Nevertheless, this was the first report of metformin-related B12 deficiency from this region.

## Conclusions

This study observed that metformin-treated patients with PCOS have lower serum vitamin B12 levels than newly diagnosed, drug-naïve ones. One-fourth of the new cases of PCOS have subnormal B12 levels; the frequency is 57.7% in metformin receivers. Further large-scale data are needed to recommend routine periodic screening for B12 levels in PCOS.
